# Survey dataset of Malaysian perception on rising cost of living

**DOI:** 10.1016/j.dib.2019.104910

**Published:** 2019-11-28

**Authors:** Nor Fatimah Che Sulaiman, Nur Azura Sanusi, Suriyani Muhamad

**Affiliations:** a Faculty of Business, Economics and Social Development, Universiti Malaysia, Terengganu, Malaysia; b Institute of Tropical Biodiversity and Sustainable Development, Universiti Malaysia, Terengganu, Malaysia

**Keywords:** Household, Income, Consumption expenditure, Standard of living

## Abstract

The introduction of good and services tax (GST) that has replaced the sales and services tax (SST) had contributed to the rising cost of living in Malaysia. The focus of this research was to present a data article on the response and perception of Malaysian households about the increasing cost of living. A descriptive research design was adopted in this study. Data were obtained from randomly selected 751 respondents of households across Malaysia. The data were collected through a structured questionnaire. Data analysis was carried out using tables and percentages. The findings show the negative perceptions of Malaysian households on the increase in the cost of living. There are various causes of the rising cost of living and can be inferred based on the perspective of income changes, price changes and patterns household consumption expenditure.

**Table:** Specifications Table

Subject	Economics
Specific subject area	Economic Development
Type of data	Table Figure Text
How data were acquired	Survey
Data format	Raw Analysed Descriptive Statistical
Parameters for data collection	Income, price and household consumption expenditure
Description of data collection	Data were gained through questionnaires using stratified random sampling. Questionnaires were screened manually for missing values or irrelevant values before the data analysis. Reliability test applied before analysis.
Data source location	All states in Malaysia; Johor, Kedah, Kelantan, Melaka, Negeri Sembilan, Pahang, Pulau Pinang, Perak, Perlis, Selangor, Terengganu, Sabah, Sarawak and Wilayah Persekutuan Kuala Lumpur.
Data accessibility	All the data are in this data article as a supplementary data file.
Related research article	Che Sulaiman N.F., Economic Growth, Income Distribution and Development of Inclusive Growth Index, (Ph.D. thesis), Universiti Kebangsaan Malaysia, Bangi, 2018 [1].

**Table:** 

**Value of the Data** • The data will be useful to analyze the response and perception of Malaysian households about the increasing cost of living and other comparable countries having the same features and situation. • The data is valuable for further research to formulate the strategic program on poverty alleviation and increase the standard of living. • The data can be used by policy makers and researchers to understand the importance of the interrelationship between incomes, price and consumption expenditure of households towards attaining a better standard of living [2, 3].

## 1 Data

The survey has been carried out through a public questionnaire conducted simultaneously throughout the country. The objective of the questionnaire was to collect feedback and perceptions of the community on the rising cost of living. A total of 751 respondents were interviewed and responded to questionnaires distributed. Selangor had the highest number of respondents, of which 109 were followed by Sarawak and Perak. On average, each state represented more than 40 survey respondents (see Table 1, Table 2).

**Table 1:** Respondents by state.

State	Total Respondent
Perlis	30
Kedah	54
Pulau Pinang	47
Perak	77
Selangor	109
Kuala Lumpur & Putrajaya	44
Sarawak	79
Negeri Sembilan	30
Melaka	25
Pahang	41
Johor	73
Kelantan	45
Terengganu	28
Sabah	69

**Table 2:** Classification of respondents by locality and income group.

Area	Frequency	Percentage	Income Group	Frequency	Percentage
Urban	433	57.6%	B40	396	53%
Rural	318	42.3%	M40	245	33%
			T20	110	15%
Total	751	100%	Total	751	100%

Households in Malaysia have been divided into three different income groups. Top 20% (T20) Middle 40% and Bottom 40% (B40). The definition of T20, M40, and B40 are based on the Department of Statistics Malaysia (DOSM, 2014) and the level of income for every group has increased throughout the years; indicating economic growth. According to the Household Income and Basic Amenity Survey 2014 by DOSM, the T20 (top 20%) income group is the household that has household income above RM8,319 (USD2,377). The M40 (middle 40%) income groups have household income ranging between RM3,856 (USD1,102) and RM8,318 (USD2,376). Meanwhile, B40 (bottom 40%) income groups are the household earning monthly income below RM3,855 (USD1,101) [4].

This data also can contribute to strengthen data readiness and filling data gaps to develop a comprehensive dataset for Sustainable Development Goal (SDG) implementation by 2030. Malaysia is looking forward to achieving No Poverty (SDG Goal 1). This goal aims to end poverty in all its forms everywhere by creating sound policy frameworks at the national, regional and international levels, to support accelerated investment in poverty eradication actions [5]. Moreover, monitoring low inflation and a comfortable standard of living will ensure Malaysia would achieve the SDG 2030 of equity of economic growth and equal opportunity for all Malaysian regardless their gender and locality.

The urban population represented about 57.6% of the survey respondents while 42.3% of the respondents were rural residents. In terms of income status, the bottom 40% income group (B40) was the highest among the respondents with the highest percentage of 53% followed by the middle 40% income group (M40) by 33% and the top 20% income group (T20) by 15%.

In general, 82.3% of respondents have argued that the cost of living has increased. 354 respondents who agreed were urban residents while the other 265 respondents were rural residents. Meanwhile, there are only a small number of urban and rural populations who do not agree that the present cost of living has increased. Therefore, 81.8% of the urban population and 83.6% of the rural population have voiced their concern about the rising cost of living [6]. The perception of the rising cost of living by income group also showed the same trend. Nearly all B40 income group (83.6%) agreed with the rising cost of living that has taken place. In fact, the majority of the T20 income group (78.2%) also expressed anxiety about the rising cost of living despite their relatively lucrative income [7] (see Table 3).

Furthermore, from 620 respondents who claimed that cost of living had increased, 29.2% of respondents felt that GST was the reason of the rising cost of living. Meanwhile, 60.9% of respondents claimed that the price hikes of goods and services were the cause of rising cost of living. Only 4.4% of respondents stated that low-paid salary lead to rising cost of living. Table 4 shows respondents' perceptions of the causes of rising cost of living.

**Table 3:** Distribution of respondent perception on the rising cost of living by area and income group.

Area	Yes	No	Total	Income group	Yes	No	Total
Urban	354	79	433	B40	331	65	396
Rural	266	52	318	M40	203	42	245
				T20	86	24	110
Total	620	131	751	Total	620	131	751

**Table 4:** Malaysian perception of the rising cost of living.

Reason	Number of Respondents	Percentage of Respondents^a^
GST	181	29.2%
Price Hike	377	60.9%
Low Salary	27	4.4%

a From a total of 620 respondents who agreed.

## 2 Experimental design, materials, and methods

**Fig. 1:**
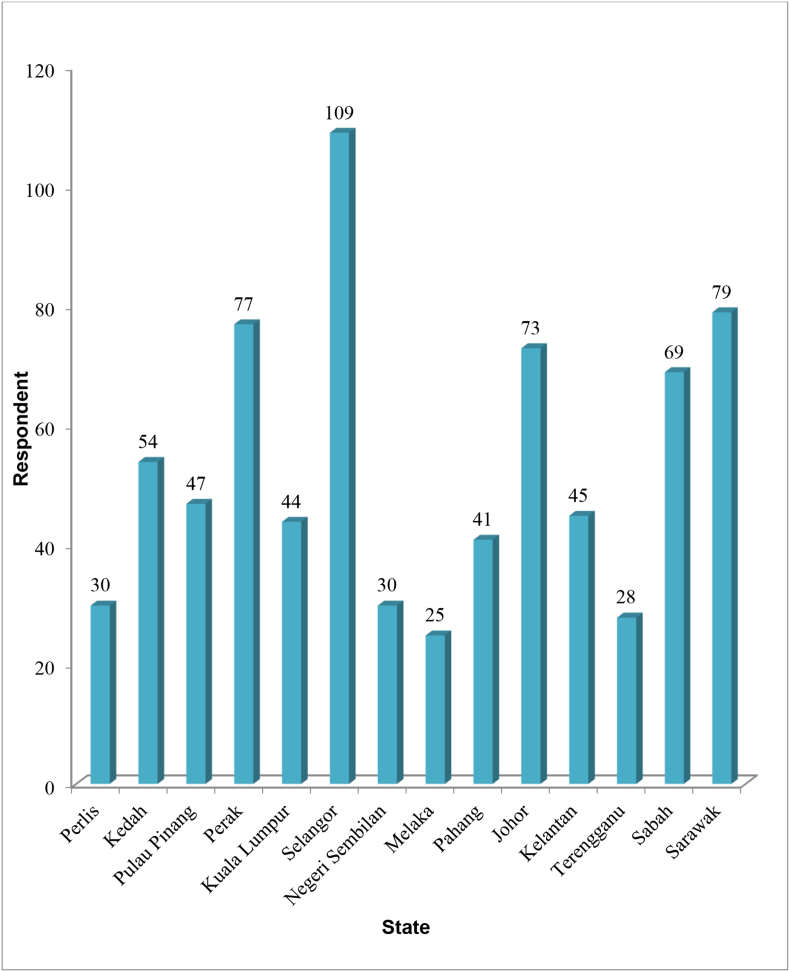
Respondents by state.

The researcher adopted a survey research design to obtain data from 751 respondents from 14 states in Malaysia. All states in Malaysia are Johor, Kedah, Kelantan, Melaka, Negeri Sembilan, Pahang, Pulau Pinang, Perak, Perlis, Selangor, Terengganu, Sabah, Sarawak, and Wilayah Persekutuan Kuala Lumpur. Data were gathered by means of a structured questionnaire (Appendix 1). The questionnaire was divided into several sections. Section 1 was used to obtain demographic information from respondents. Section 2 assessed the economic status of the respondents. Section 3 and 4 gathered information about household income and assets ownership. Section 5 was used to obtain household consumption expenditure and last section Section 6 assessed the information about perception of Malaysian households about rising cost of living [8]. The data were qualitatively analysed and presented in tables (1–5) and Fig. 1. Ethical consideration in the research process was ensured because administering the questionnaires to respondents was based on their willingness to respond to the research instrument.
