# Supplementary material for: Survey dataset of Malaysian perception on rising cost of living
Source: Data Brief. 2019 Nov 28;28:104910. doi: 10.1016/j.dib.2019.104910 (PMC6926134; doi:10.1016/j.dib.2019.104910)
Supplement: Multimedia component 1 [file mmc1.pdf]

PRIVATE AND CONFIDENTIAL

COST OF LIVING RESEARCH

Category: Household	QUESTIONNAIRE	Respondent Code: ________

Survey Information	
Date	
Time	from _______am/pm to ________ am/pm
Location (ect: Bandar Baru Bangi, Selangor)	
Enumerator name	
Reviewed by & Review Date	

PART 1: RESPONDENT BACKGROUND

1.	Name	
2.	I/C No (New I/C)	
3.	District	
4.	Urban/Rural	
5.	Tel No. /Hp. No	

7. Gender: □ Male □ Female

8. Age: ______

9. Educational status:
- □ No Education
- □ With Education, the Highest Education Stage:
  - a) Formal Education:
    - □ Primary school drop out
    - □ Finished primary school
    - □ Form 3 / SRP/ PMR / PT3 / Skill certificate (specify): ____________________
    - □ Form 5 / O Level / SPM / Skill certificate (specify): ____________________
    - □ Form 6 / A Level/ STPM/ Matriculation
    - □ Diploma
    - □ Bachelor degree
    - □ Master degree
    - □ Philosophy Doctor
  - b) Informal education
    - □ Specify: _____________________________________

10. Marital Status:
- □ Single
- □ Married
- □ Divorced

11. What is your health status?
- □ Healthy
- □ Not healthy (specify):_________________________________)

12. Number of children (if any): ____.

13. Number of household: __________

14. Are you the Head of Household (HoH)? □ Yes □ No

15. Number of dependents (if any): _______

PART 2: ECONOMIC STATUS

1. Do respondents have a job?
   □ Yes, specify type of job: ___________________________________
   □ No, looking for a job?
      □ Yes (length of unemployment, ___________________________ month)
      □No (Reason:____)

(If you answered No, go to Question 4)

2. Do you change your job?
   □ Yes, How often do you change jobs? ____________________ in last year
   Why change jobs? ____
   □ No.

3. Total individual monthly income: RM_____________

4. Information on the amount of tax paid

No	Type of tax	Monthly (RM)	Yearly (RM)
a.	Income tax		
b.	Assessment rates		
c.	Quit rent		
d.	Others, specify: ______________________		

PART 3: INCOME INFORMATION

1. Indicate the monthly income of households (head of household and household members) from paid employment sources.

Type of salary income	(A) Head of Household (RM monthly)	Working household members (living and dining together) (RM monthly)					(B) Total household income (RM)
		1	2	3	4	5	
Gross income (public sector)							
Gross income (private sector)							
Income from self-employement							
Pension							
Cash overtime income, commissions etc.							
Others (e.g part time job) (If any, please specify) …………………….........................							
**TOTAL (RM)**							

2. Indicate the income (if any) of the transfer / contribution.

Type of transfer income	(A) Head of Household (RM monthly)	Working household members (living and dining together) (RM monthly)					(B) Total household income (RM)
		1	2	3	4	5	
Contribution from other household members							
Social welfare assistance – Social Welfare Department							
Zakat							
Other cash assistance. (If any: specify)... ……………………………………… ……………………...….………….							
**TOTAL (RM)**							

3. Income from property (if any)

Type of property income	(B)<br>Household and total household income (RM)
Agricultural land rental income (including farmland / farm lease)	
Rent from houses or other property	
Rental equipment (boats, nets, machinery, cars etc)	
Dividends (Cooperation and other shares)	
Other property income. (specify) ....<br>………………..<br>………………..	
**TOTAL (RM)**	

PART 4: ASSET INFORMATION

No	Assets / properties owned	No. Unit / Total Area	Payment status		Activities (personal / rental use, taxes, agriculture, business etc)	Revenue from assets (RM)
			Yes	No		
Real estate						
1.	Land					
2.	House					
3.	Building / Shop / Factory					
Vehicle						
1.	Car					
2.	Van					
3.	Motocycle					
Equipment						
1.	Machinery					
2.	Boat					
3.	Boat engine					
4.	Business permit / license					
5.	Others. Please specify:<br>…………………………<br>…………………………<br>…………………………					

PART 5: MONTHLY EXPENSES INFORMATION

1. Please indicate where and how often you spend for your daily needs:

No	Place	Frequency per Week
1.	Night market / farmers market	0 1 2 3 4 5 6 7
2.	Grocery store	0 1 2 3 4 5 6 7
3	Kedai Rakyat 1 Malaysia	0 1 2 3 4 5 6 7
4.	Supermarkets	0 1 2 3 4 5 6 7
5.	Others. Please specify: ______________	0 1 2 3 4 5 6 7

2. Do you have debt?

□ No

□ Yes, specify:

No.	Type of debt	Monthly payment (RM)
a.	Credit card	
b.	Car hire purchase installment	
c.	Motocycle hire purchase installment	
d.	Housing loan installment	
e.	Educational loan	
f.	Personal loan	
g.	Input loan (agricultural, industrial etc)	
h.	Other loans (purchases of furniture, home appliances, etc.)<br>Please specify,<br>………………………………………………………..<br>……………………………………………………….<br>……………………………………………………….	

3. Total (or monthly) savings in the bank / house? RM __________________
(Please declare 12 months if monthly savings)

4. . Do you subscribe to an insurance scheme?

□ Yes, type of insurance ___________ ((Examples: Life Insurance, Vehicles, Health, Accidents)

□ Not subscribed, because ____

5. Monthly expenses:

Type of monthly expenses	Total (RM)
(i) Kitchen/daily	
(ii) Dine outside	
(iii) House rental	
(iv) Maintenance (home, vehicle, sewage)	
(v) Education	
(vi) Caregiver / Maid	
(vii) Water	
(viii) Electricity	
(ix) Astro	
(x) Internet – Unifi / Streamyx	
(xi) Telephone/Handphone	
(xii) Transportation (petrol)	
(xiii) Transportation Fares (Buses, Train, Boats)	
a. Bus	
b. Train (KTM/LRT)	
c. Boat	
(xiv) Health/ Medical	
(xv) Insurance (Health/Accident)	
(xvi) Loan repayment	
a.Housing loan	
b.Car loan	

c.Motorcycle loan	
d.Education	
e.Others, please specify ________________________	
(xvii) School, school bus fares, tuition	
(xviii) Clothing	
(xix) Recreation/leisure	
(xx) Others, please specify ___________________	
TOTAL MONTHLY EXPENDITURE	RM
TOTAL MONTHLY NET INCOME	RM
TOTAL YEARLY NET INCOME (# X 12)	RM

6. Has the cost of living increased over the last 6 months?

a) No.

b) Yes, Why do you say that?

____

____

PART 6: HOUSEHOLD PERCEPTION OF INCREASE IN PRICE OF GOODS

Instructions: Circle the following questions on a Likert scale of 1-5, 1 = strongly disagree and 5 = strongly agree

A. KNOWLEDGE

Circle the following questions on a Likert scale of 1-5, 1 = most disagree and 5 = most agree

I think the following factors have led to the rise in prices of goods	Feedback
1) Increased household income led to higher prices of goods	1 2 3 4 5
2) Retailers are always taking advantage	1 2 3 4 5
3) Production costs have increased. The price of goods also has to rise.	1 2 3 4 5
4) The transportion cost led to the increase in prices of goods.	1 2 3 4 5
5) Middle man cause the price of goods to be more expensive.	1 2 3 4 5
6) The household lifestyle who like to spend causes an increase in prices of goods.	1 2 3 4 5
7) The implementation of GST caused price hikes	1 2 3 4 5
8) Climate change such as rainy season causes price hikes	1 2 3 4 5
9) During the festive season, retailers increase prices to maximize profits.	1 2 3 4 5

B. PRACTICE

Circle the following questions on a Likert scale of 1-5, 1 = strongly disagree and 5 = strongly agree

Statement	Feedback
1) I buy items within my budget	1 2 3 4 5
2) I buy more goods during the festive season even though it's expensive	1 2 3 4 5
3) I love to dine outside.	1 2 3 4 5
4) I only buy what is needed even when there is a discount for less essential items.	1 2 3 4 5
5) I often bring lunch from home	1 2 3 4 5
6) I usually use credit card to buy groceries	1 2 3 4 5
7) I often buy goods from wholesale or wholesale markets.	1 2 3 4 5
8) I use public transport to work / school	1 2 3 4 5
9) In the event of a shortage of goods, I will collect them.	1 2 3 4 5

PART 7: HOUSEHOLD SUGGESTION IN ADDRESSING HIGHER COST OF LIVING

1. In my opinion, the following government strategies should be pursued as they help reduce the cost of living:

No.	Policies / Initiatives / Strategies	Continue/ Discontinue/No idea	Reason
1.	Bantuan Rakyat 1 Malaysia (BR1M)		
2.	e-Kasih (1AZAM)		
3.	Perumahan Rakyat 1 Malaysia (PR1MA)		
4.	Bantuan Buku 1 Malaysia (BB1M)		
5.	Klinik 1 Malaysia		
6.	Kedai 1 Malaysia		
7.	Skim Latihan 1 Malaysia (SL1M)		

1. The following are suggestions for reducing the cost of living:

a) Government :

b) Producer:

c) Household:

THANK YOU
